# Effects of Late Gestational Fetal Exposure to Dexamethasone Administration on the Postnatal Hypothalamus-Pituitary-Adrenal Axis Response to Hypoglycemia in Pigs

**DOI:** 10.3390/ijms18112241

**Published:** 2017-10-27

**Authors:** René Schiffner, Guadalupe L. Rodríguez-González, Florian Rakers, Marius Nistor, Peter W. Nathanielsz, Teodora Daneva, Matthias Schwab, Thomas Lehmann, Martin Schmidt

**Affiliations:** 1 Department of Neurology, Jena University Hospital—Friedrich Schiller University, 07747 Jena, Germany; Florian.Rakers@med.uni-jena.de (F.R.); vorenus@web.de (M.N.); Matthias.Schwab@med.uni-jena.de (M.S.); 2 Orthopaedic Department, Jena University Hospital—Friedrich Schiller University, 07747 Jena, Germany; 3 Reproductive Biology, National Institute of Medical Science and Nutrition, 14000 Mexico City, Mexico; letyrodgon@hotmail.com; 4 Department of Animal Science, University of Wyoming, Laramie, 82071 WY, USA; Peter.Nathanielsz@uwyo.edu; 5 Institute of Biology and Immunology of Reproduction, Bulgarian Academy of Sciences, 1113 Sofia, Bulgaria; danevadoki@abv.bg; 6 Institute of Medical Statistics, Computer Sciences and Documentation Science, Jena University Hospital—Friedrich Schiller University, 07743 Jena, Germany; Thomas.Lehmann@med.uni-jena.de; 7 Institute for Biochemistry II, Jena University Hospital—Friedrich Schiller University, 07743 Jena, Germany; Martin.Schmidt@med.uni-jena.de

**Keywords:** HPA axis, ACTH, cortisol, stress response, hypoglycemia

## Abstract

Background: Prenatal glucocorticoid administration alters the activity of the fetal hypothalamic-pituitary-adrenocortical axis (HPAA), and correspondingly the adenocorticotropic hormone (ACTH) and cortisol levels after birth. The dosages required for these effects are critically discussed. Activation of the HPAA is related to metabolic syndrome and diabetes mellitus. Hypoglycemia is the classic side effect of antidiabetic treatment. We hypothesized that a low dosage of dexamethasone in late pregnancy alters the HPAA response to hypoglycemia in pigs. Methods: 12 pregnant sows were randomly assigned to two groups which received either a low-dose intramuscular injection (99th and 100th day of gestation) of dexamethasone (0.06 μg/kg body weight) or vehicle. Three months after birth, 18 dexamethasone-treated anaesthetized offspring and 12 control offspring underwent a 75 min hypoglycemic clamp (blood glucose below 4 mmol/L) procedure. Heart rate (HR), blood pressure, ACTH and cortisol levels and body weight (at birth and after three months) were recorded. Results: Dexamethasone-treated animals exhibited significantly elevated ACTH (139.9 ± 12.7 pg/mL) and cortisol (483.1 ± 30.3 nmol/L) levels during hypoglycemia as compared to the control group (41.7 ± 6.5 pg/mL and 257.9 ± 26.7 nmol/L, respectively), as well as an elevated HR (205.5 ± 5.7 bpm) and blood pressure (systolic: 128.6 ± 1.5, diastolic: 85.7 ± 0.7 mmHg) response as compared to the control group (153.2 ± 4.5 bpm; systolic: 118.6 ± 1.6, diastolic: 79.5 ± 1.4 mmHg, respectively; *p* < 0.001). Conclusions: Low-dose prenatal administration of dexamethasone not only exerts effects on the HPAA (ACTH and cortisol concentration) and vital parameters (HR and diastolic blood pressure) under baseline conditions, but also on ACTH, HR and systolic blood pressure during hypoglycemia.

## 1. Introduction

Endogenous gluconeogenesis is important for the maintenance of a stable blood glucose homeostasis in both humans and pigs [1]. Activation of the sympathetic nervous system may be related to metabolic syndrome and disorders of glucose metabolism, e.g., diabetes mellitus [2,3,4,5]. Previous studies have already provided solid evidence that prenatal maternal injection of synthetic glucocorticoids (GCs) [6,7,8], such as betamethasone and dexamethasone, induces changes in the activity of the hypothalamic-pituitary-adrenocortical axis (HPAA), which is responsible for the distribution of endogenous catecholamines. Therefore, excessive exposure to GCs during gestation may have an adverse impact on stress responsive systems [9]. Changes in the HPAA have an influence on endogenous stress hormones like the adenocorticotropic hormone (ACTH) and cortisol, which stimulate glucose release [10,11], as well as on the autonomous nervous system, which is involved in gluconeogenesis [12,13]. Hypoglycemia, with a resulting increase of the endogenous stress hormones ACTH and cortisol, is a classic side effect of antidiabetic treatment, e.g., inulin or oral antidiabetic drugs (sulfonylureas). Prenatal maternal GC injections in late pregnancy have a direct influence on changes in HPAA in both humans and pigs [14,15,16]. The exact dosages of injected GCs required for such an alteration remain controversial. 

We tested the hypothesis that a prenatal maternal low dosage injection of dexamethasone would cause an alteration of the counteregulatory responses of endogenous ACTH and cortisol during hypoglycemia in pigs. Severe hypoglycemia causes a HPAA response that is expressed as enhanced endogenous ACTH and cortisol concentrations. We decided to use the GC dexamethasone since it has a higher affinity for glucocorticoid receptors than betamethasone. The repeated injections of dexamethasone, at the 99th and 100th day of gestation (dGA) in a 12 h interval, were designed to provoke a distinct response [17,18,19,20,21]. The experimental setup allows the experiment to be performed under deep general anesthesia. 

## 2. Results

The experimental setup was appropriate to detect differences between offspring of dexamethasone-treated pigs and non-treated pigs with respect to body weight and growth over the course of the observation period (Table 1). No gender-specific differences in body weight and growth were observed during the observation period.

**Figure 1:**
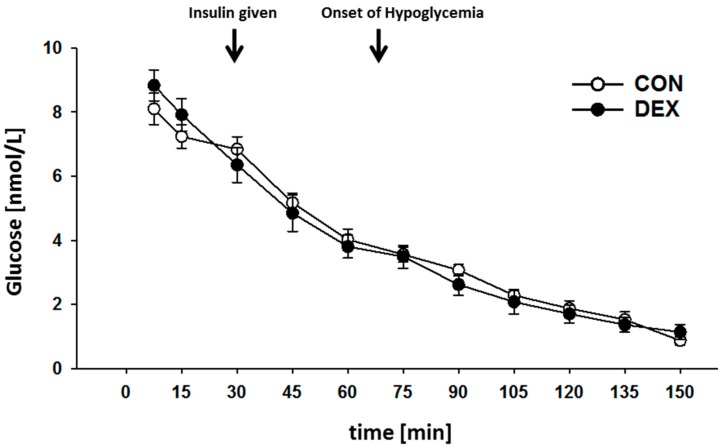
Blood glucose levels during hypoglycemic clamp. Blood glucose (mmol/L) measurements were performed every 15 min, starting with the anesthesia. Application of insulin was after the 30 min of baseline. The arrows mark the first insulin injection and the start of the hypoglycemic period. Data are given as mean ± SEM for the control group (CON) and the dexamethasone-treated group (DEX).

Hypoglycemia was considered to be established at blood glucose concentrations below 4 mmol/L and was maintained for 75 min (Figure 1). Baseline arterial blood glucose concentrations did not differ between both groups and were 8.1 ± 0.4 mmol/L in controls and 8.8 ± 0.4 mmol/L in the dexamethasone-treated group. At the end of the observation period, i.e., after 150 min and the hypoglycemic clamp procedure, the arterial blood glucose concentration was 0.9 ± 0.1 mmol/L in the control group and 1.1 ± 0.2 mmol/L in the dexamethasone-treated group. As the effective time of an established hypoglycemia differed slightly between individual animals and the two groups, all subsequent measurements in each individual animal refer to the first sampling in which the blood glucose was below the threshold of 4 mmol/L.

**Figure 2:**
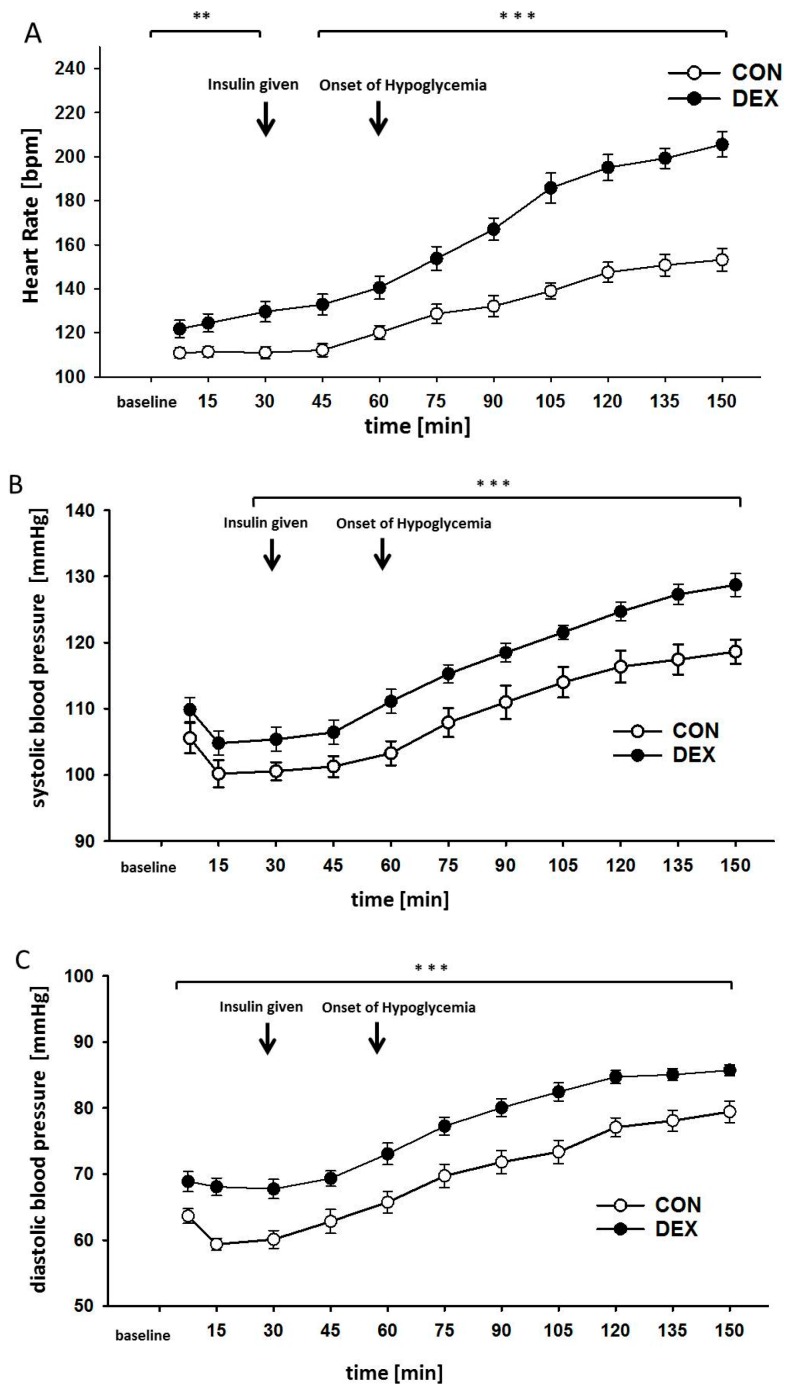
Effects of hypoglycemia on vital parameters. (**A**) Heart rate; (**B**) systolic and (**C**) diastolic arterial blood pressures. Data are given as mean ± SEM for the control group (CON) and the dexamethasone-treated group (DEX), ** *p* < 0.03, *** *p* < 0.001.

During normoglycemic baseline conditions, heart rate (HR) and systolic and diastolic blood pressures were higher in the dexamethasone-treated group than in the control group (*p* < 0.05), and furthermore, remained higher over the course of the entire hypoglycemic period (*p* < 0.001, Figure 2). At baseline, the HR of the dexamethasone-treated group was significantly higher than that of the control group (mean difference = 10.9 bpm, *p* = 0.033). Only the HR of the dexamethasone-treated group increased significantly during anesthesia, on average by 0.3 bpm per minute (*p* < 0.001). No significant differences between the systolic blood pressures of the groups were observed at baseline. The diastolic blood pressure in the dexamethasone-treated group was significantly higher than in the control group at baseline (mean difference = 6.4 mmHg, *p* = 0.009). Significant differences of diastolic blood pressure existed over the course of baseline between the groups. 

During the hypoglycemic clamp, a rapid increase of HR and systolic and diastolic blood pressures could be observed in the dexamethasone-treated group. The HR in the dexamethasone-treated group was significantly higher than in the control group during hypoglycemia (mean difference = 22.6 bpm, *p* < 0.001). During the hypoglycemic clamp, a significant increase in the HR by 0.8 bpm was observed in the dexamethasone-treated group per minute (*p* < 0.001), while an increase of 0.4 bpm per minute (*p* < 0.001) was observed in the control group. The systolic blood pressure was significantly higher in the dexamethasone-treated group as compared to the control group (mean difference = 8.3 mmHg, *p* = 0.01). The dexamethasone-treated group exhibited an increase of the systolic blood pressure of 0.19 mmHg per minute (*p* < 0.01), as compared to an increase of. 0.17 mmHg per minute in the control group (*p* < 0.05). Significant differences could also be observed with respect to the diastolic blood pressure at baseline, which was higher in the dexamethasone-treated group (mean difference = 8.2 mmHG, *p* < 0.001); although the dexamethasone-treated group, with 0.14 mmHg per minute, did not exhibit a significantly higher increase of the diastolic blood pressure during hypoglycemia as compared to the control group with. 0.13 mmHg per minute (*p* > 0.05). Significant difference between the groups with respect to heart rate and systolic and diastolic blood pressures over the course of the observation period (*p* < 0.001, Figure 2). No gender specific differences were observed with regard to HR and systolic and diastolic blood pressures.

**Figure 3:**
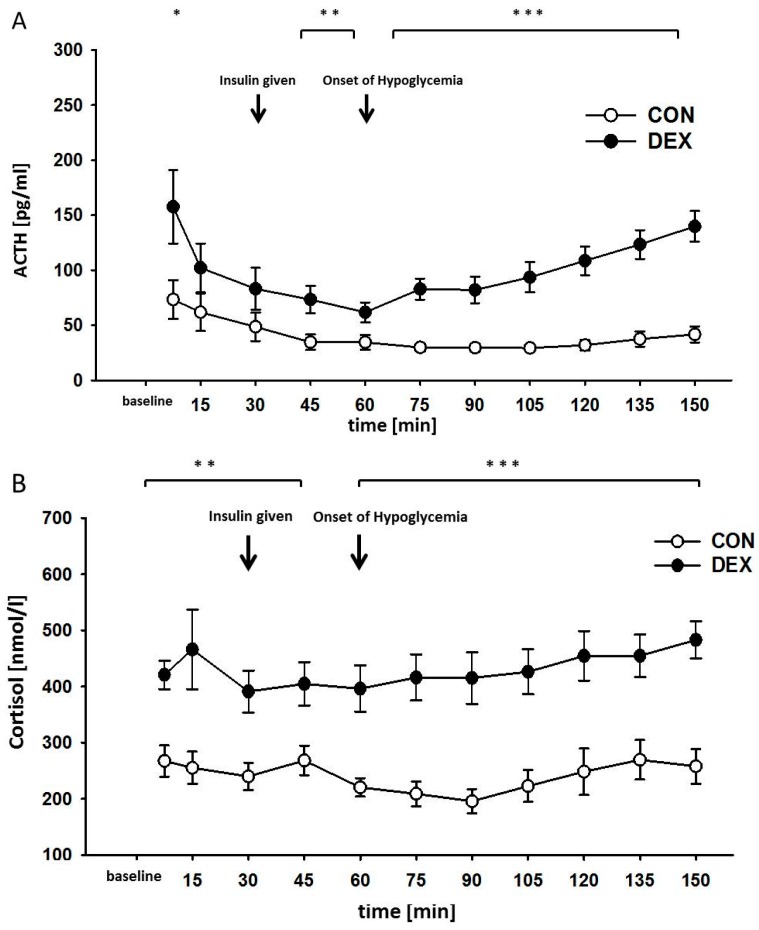
ACTH (**A**) and cortisol (**B**) plasma concentrations during hypoglycemic clamp. Data are given as means ± SEM for the control group (CON) and the dexamethasone-treated group (DEX), * *p* < 0.05, ** *p* < 0.03, *** *p* < 0.001.

Under both normoglycemic and hypoglycemic conditions, different concentrations of serum ACTH and cortisol were recorded for both groups over the course of 11 consecutive measurements, performed every 15 min (Figure 3A,B). At baseline, the ACTH concentration of the control group was significantly lower than that of the dexamethasone-treated group (mean difference = 69.1 pg/mL, *p* = 0.038). While the ACTH concentration decreased in both groups during anesthesia and surgical intervention, a more marked decrease was observed in the dexamethasone-treated group. While the control group exhibited a decrease of 0.9 pg/mL per minute (*p* < 0.05), a decrease of 1.5 pg/mL per minute was measured in the dexamethasone group (*p* < 0.001). 

The increase in ACTH concentration per minute differed in both groups, a stronger increase of 0.6 pg/mL per minute was measured in the dexamethasone-treated group (*p* < 0.001), while the control group exhibited an. of 0.1 pg/mL per minute during hypoglycemia (*p* < 0.001).

The absolute concentration differed between both groups during the observation period (*p* < 0.001, Figure 3A). Cortisol levels were increased during anesthesia and hypoglycemia in the dexamethasone-treated group. Under normoglycemic conditions, we measured a decrease in ACTH concentration in the control group from 73.3 ± 15.6 to 34.6 ± 6.3 pg/mL, while a decrease from 157.6 ± 30.5 to 73.3 ± 11.4 pg/mL was recorded in the dexamethasone-treated group. During the induced hypoglycemia, we observed an increase in ACTH concentration in both groups, albeit to a different extent. In the control group, we measured an increase in ACTH concentration to 41.7 ± 6.5 pg/mL, while in the dexamethasone-treated group an increase to 139.9 ± 12.7 pg/mL was observed under hypoglycemic conditions. The course of the cortisol concentration differed between the two groups as well (*p* < 0.001, Figure 3B). At baseline, the cortisol concentration was significantly lower in the control group than in the dexamethasone-treated group (mean difference = 168.3 nmol/mL, *p* = 0.013). As compared to the baseline value of 267.3 ± 23.7 nmol/mL, the control group remained nearly unchanged under normoglycemic anesthesia (268.3 ± 22.5 nmol/mL) as well as hypoglycemia (257.9 ± 26.7 nmol/mL). In contrast, the dexamethasone-treated group exhibited a decrease from baseline 420.9 ± 23.1 to 404.9 ± 35.3 nmol/mL under normoglycemic conditions, with a subsequent increase to 483.1 ± 30.3 nmol/mL under hypoglycemia at the end of the observation period. On average, hypoglycemia significantly increased the cortisol concentration by 1.2 nmol/mL per minute (*p* < 0.001) in the dexamethasone-treated group (Figure 3B).

No gender specific differences with regard to ACTH and cortisol concentrations could be observed over the course of the observation period. 

## 3. Discussion

In pigs, a late gestational dexamethasone injection causes altered levels of the stress hormones ACTH and cortisol in the next generation.

To test our hypothesis that ACTH and cortisol concentrations are altered in specimens that received a prenatal injection of dexamethasone, we utilized pigs as an experimental model. During both anesthesia and surgical intervention under normoglycemic conditions, and under an induced severe hypoglycemia, we were able to observe differing courses of ACTH and cortisol concentrations between the two experimental groups. We observed both birth weight and growth restrictions in the dexamethasone-treated group, consistent with results of similar studies in humans and pigs [18,19,20,22]. The pigs utilized in our experiment were healthy and received no drugs that affect blood pressure, liver or kidney function or glucose metabolism (for example catecholamines), prior to the experiments. The functional significance of the HPAA has recently attracted much attention in this field of research. Both ACTH and cortisol constitute hormonal responses of the HPAA [23,24]. As the end product of the HPAA, cortisol is increased in the circulation in response to enhanced levels of ACTH. Both ACTH and cortisol are essential components of the endogenous system for the maintenance of energy homeostasis. In addition, this study and other recent investigations demonstrate that the HPAA and the autonomous nervous system have a key role in transmitting metabolic information [13]. The increased levels of ACTH and cortisol can be explained in the context of surgical preparation and anesthesia. Due to this stress response, the initial blood glucose concentration decreased in both groups. A moderate decrease of ACTH and cortisol levels, as well as of blood glucose concentration is a well-known effect of anesthesia [25,26,27], which was observed during our experiments in both groups as well. A correlation between surgical preparation, as well as anesthesia, and blood glucose level is similarly known to exist [25,26,27]. During the hypoglycemic clamp, the only difference between groups with regard to an increased endogenous stress hormone is represented by the observed increase of ACTH concentration. The hypothalamus in particular is a primary site of convergence and integration of redundant energy status signaling, which encompasses both central and peripheral neural inputs, as well as hormonal and nutritional factors [28]. These inter-tissue communication pathways are based on circulating humoral factors, including insulin and adipocytokines. These peptides also function outside the central nervous system by influencing the activities of neurons, e.g., the vagal afferent nerve that projects to the nucleus of the solitary tract in the brainstem [29]. Since ACTH and cortisol are influenced by the circadian rhythm [30,31], all experiments were conducted at the same time, which allowed us to assume a uniform circadian rhythm in all pigs.

We decided to use the GC dexamethasone for this study due to its higher affinity for glucocorticoid receptors. Furthermore, we administered a repeated dose of dexamethasone, at the 99th and 100th dGA in a 12 h interval, to provoke a more distinct response [17,18,19,20,21]. However, we utilized a reduced single dose of 0.06 mg/kg body weight to verify that even smaller doses of GCs would correspond to altered levels of stress hormones as described above. The usual human dosage is 6 mg dexamethasone per patient, which corresponds to 0.075 mg/kg body weight dexramethasone in an 80 kg patient [32].

The pathophysiological correlate of our experimental model of severe hypoglycemia in humans is not the well managed diabetic patient, or the patient with an elevated blood glucose level. The experimental setting of an induced hypoglycemia mimics an acute complication in patients with diabetes mellitus (both type 1 and type 2), which results from an overdose of insulin, some other antidiabetic drugs or stress. Therefore, we applied severe hypoglycemia with blood glucose levels that are often observed in patients affected in a similar way. In order to avoid a too rapid decrease of blood glucose level throughout the course of the experiment, we induced hypoglycemia via repetitive administrations of insulin. In pilot tests for the experimental model of this study, we established hypoglycemia with a stable blood glucose concentration of 1.8 mmol/L in three animals. Unfortunately, it quickly became apparent that a steady administration of insulin and glucose (40% via perfusors) causes high variations in the glucose metabolisation and a decrease of ACTH and cortisol concentrations. The created acute situation serves to demonstrate the involvement of the HPAA during hypoglycemia and anaesthesia. Even taking into account the extreme conditions utilized, the experimental setting ensured reproducibility of the experiment.

One limitation of our study is that, although we measured ACTH and cortisol concentrations, we did not record the heart beat variability during the experiment. Heart beat variability examines whether the interaction between cardio-autonomic control and HPA axis activity better explains perceived stress responses [33]. Unlike other studies, the focus of our study was to investigate the stress response, not heart beat variability. During hypoglycemia, the glucose metabolism is primarily mediated through stress responses (endogenous hormone release of ACTH and cortisol). The heart beat variability can be considered as an end-organ specific reaction to stress [34,35]. Therefore, although dexamethasone injection during pregnancy is a typical procedure that induces changes in the HPAA, this study can only demonstrate an alteration of the HPAA response but not a stress axis alteration per se. Although we have utilized only a relatively low number of sows, the advantage of this experimental model lies in the reduced number of test animals. Although we were not able to identify any gender-specific differences, this might be due to the early castration in the agricultural holding facility.

In our estimate, a direct transfer of our findings on humans is possible, especially since alterations of the HPAA are already described in serious studies, and long-term chronical alterations can be found in patients that received prenatal dexamethasone treatment. Furthermore, baseline concentrations of both ACTH and cortisol are similar under stress conditions in humans and pigs [36].

## 4. Methods

### 4.1. Breeding and Experimental Groups

The experiment was performed in strict accordance to local standards and the “Guide for the Care and Use of Laboratory Animals” [37]. All procedures were approved by the Saxony animal welfare committee (Leipzig; permission number: TVV 07/13 valid from 29 April 2013 until 30 April 2018). Female pregnant German land race pigs were mated in a conventional agricultural holding for rearing pigs (for mainstream food industry, 35,000 pigs per year), which delivers time-dated pregnant sows. We randomly assigned the 12 female pregnant pigs in to two groups. The pregnant sows either received an intramuscular injection of dexamethasone (Dexamethason Injektionslösung ad us. vet^®^, CP-Pharma, Burgdorf, Germany), (60 μg/kg body weight, *n* = 6) on the 99th and 100th day of gestation (dGA, term = 154–156 days) with 6–10 mL stock solution or equal volume of isotonic saline (Isotonische Kochsalzlösung^®^, Fresenius, Bad Homburg, Germany) (*n* = 6). Although the clinical correlate would be a single shot therapy, application schemes remain to be either 2 × 12 mg/per 24 h betamethasone or 4 × 6 mg dexamethasone every 12 h (intramuscular injection) [38,39]. Ealier investigations have shown that two doses of betamethose were more effective in the promotion of fetal lung maturation than dexamethasone [40]. An important distinction of our study was the low dose of utilised corticosterioids. We therefore decreased the dose significantly and extended the application interval. Sows were left to deliver their offspring under normal agricultural conditions. Litter size did not differ between dexamethasone and control sows (Table 2). There were no still births. Three (dexamethasone-treated group) or two (control group) offspring of each litter were randomly chosen under consideration of sex. Animals weights of the offspring were recorded at birth and day 28 and 67 (Table 1). Male pigs were castrated on day 28 after the birth. The pigs were weaned between 67–77 days. Within the two study groups, we randomly assigned individual pigs in the following manner: 12 of 80 offspring of control pigs (CON), and 18 of 78 offspring of dexamethasone-treated pigs (DEX) were used in the subsequent experiments (Table 2).

### 4.2. Surgical Procedures

All experiments began at 09:00 a.m. Surgery, under sterile conditions and under anaesthesia, was conducted in the supine position subsequent to 24 h food withdrawal. Anaesthesia was achieved by intramuscular administration of 15 mg/kg ketamine hydrochloride (Ketavet^®^, 100 mg/mL, Pharmacia Upjohn, Erlangen, Germany) and 0.2 mg/kg midazolam hydrochloride (Midazolam-ratiopharm^®^, Ulm, Germany). Thereafter, 0.2–0.3 mg/kg propofol (Disoprivan^®^, AstraZeneca, Wedel, Germany) was administered via a 21G venous catheter (Vasocan^®^, Braun Melsungen, Melsungen, Germany) in the ear vein and the pigs were intubated orotracheally (Trachealtubus, Rüsch, Kernen, Germany). Continuous inhalation of 1.5% isoflurane (Isofluran^®^, DeltaSelect, Dreieich, Germany) and O_2_ ensured the maintainance of anaesthesia. As an analgetic, 0.003 mg/kg fentanyl per hour (0.05 mg/mL Fentanyl, Janssen) was utilized. Administration of up to 0.1 mg/kg pancuronium (Pancuronium-Actavis^®^, Actavis, München, Germany) was utilized to induce muscular relaxation. The anesthetic induction took 10–15 min in all animals. Thereafter, the animals were placed into the surgical position, intubated and all vascular accesses were established; the different procedures lasted 15–30 min in total. In order to ensure comparable preinvasive preparation times in all animals, baseline recordings began 45 min after anesthetic induction in all animals. The cornea was kept moist during anesthesia by administering eye drops (Corneregel^®^, Bausch&Lomb, Berlin, Germany). Intravenous administration of 4 mL/kg body weight isotonic saline per hour (Isotonische Kochsalzlösung^®^, Fresenius, Bad Homburg, Germany) ensured the maintenance of a controlled and stable blood pressure. The infusion therapy was terminated at the start of the baseline recordings. All animals received a comparable amount of fluid infusion, which allowed us to minimise the influence of infusions and medications on the blood pressure during the observation period. Vascular catheters (Arteriofix^®^, Braun, Melsungen, Germany) were placed into the carotid artery for blood sampling and systolic blood pressure measurement, and into the jugular vein for intraoperative administration of drugs and fluid infusion. Arterial blood pressure was recorded using transducers (Combitrans Transducer, Braun, Melsungen, Germany). Electrocardiogram (ECG) was derived using cutaneous wire electrodes. The blood pressure amplified and sampled at 1000 Hz using a data acquisition and analysis system (Labchart Pro7, ADInstruments, Spechbach, Germany). MABP was calculated and HR was triggered from R waves continuously. Then, all parameters were averaged over five seconds.

### 4.3. Hypoglycemic Clamp

The hypoglycemic clamp was induced after the 30 min of baseline recordings, utilizing a bolus of 15 IU human regular insulin (Actrapid^®^, Penfill^®^, 100 IU/mL, Novo Nordisk Pharma, Mainz, Germany) via the ear vain catheter, and was maintained between 0.5 and 4 mmol/L over 75 min by additional boluses of insulin. In order to facilitate a continually diminishing blood glucose level, additional boluses of regular insulin were administered if necessary, to mirror the clinical case of an insulin overdose. The individual total insulin dosages varied from 20 to 55 IU of insulin per experiment. A commercially available blood glucose meter (Contour^®^, Bayer AG, Leverkusen, Germany) was used to monitor the arterial blood glucose level at baseline and every 7.5 min during the hypoglycemic clamp. Arterial blood samples for ACTH and cortisol measurement were obtained at 30 min of baseline and then every 15 min. After the end of the experiments, the animals were euthanized by intravenous administration of 60 mg/kg pentobarbital (Narcoren^®^, Merial, Hallbergmoos, Germany).

### 4.4. Measurement of ACTH

ACTH was measured in EDTA plasma with a human ACTH immunoassay (ACTH ELISA, RE53081, IBL international GmbH, Hamburg, Germany). This sandwich ELISA uses antibodies binding to N-terminal and C-terminal epitopes of ACTH, respectively, which are identical in human and pig. The coefficient of variance (CV) was less than 10.3%, the inter assay CV was less than 7.1%.

### 4.5. Measurement of Cortisol

For the measurement of cortisol in EDTA plasma samples, a human cortisol immunoassay based on the competition principle (Cortisol ELISA, RE52061, IBL international GmbH, Hamburg, Germany) was used. The intra assay CV was less than 2.9% and the inter assay CV was less than 5%.

### 4.6. Statistical Analyses

Linear mixed models were fitted to analyze the difference between the two groups over time. These models ensure that the data of multiple offsprings per sow are correlated. Furthermore, the data of an offspring over time are typically not independent, which was considered in the analyses. Therefore, random effects of the sow as well as the offspring (nested in sow) were included. Fixed effects are group, gender, time and the interaction of group and time. By analyzing the interaction term we were able to test for measurement differences between the groups over time. We performed two linear mixed models for each outcome parameter, one with the data before the hypoglycemia and one during hypoglycemia.

The significance level was set to α = 0.05. All analyses were performed with IBM SPSS Statistics 23.0. Continuous outcomes are described by mean ± SEM.

## 5. Conclusions

To conclude, we were able to demonstrate that even a low-dose prenatal administration of dexamethasone causes body weight restriction, elevated responses of ACTH concentration and increased heart rate and systolic blood pressure during anesthesia and hypoglycemia. Individuals with altered HPAA and insulin-induced hypoglycemia are affected by an increased stress response. This increases the risk potential of hypoglycemia.

## Figures and Tables

**Table 1:** Weight increase in controls (CON) and prenatally dexamethasone-treated pigs (DEX) over the first three months of life.

Timepoint Heading	Control Group (CON) (kg)	Dexamethasone-Treated Group (DEX) (kg)
Birth	1.6 ± 0.02	1.3 ± 0.01 *
28 days of age	8.0 ± 0.1	6.8 ± 0.3 *
67 days of age	28.6 ± 0.2	24.1 ± 0.3 *

Data are given as mean ± SEM, * *p* < 0.001 vs. controls.

**Table 2:** Breeding data and experimental groups.

Variables	Controls (CON)	Dexamethasone-Treated (DEX)
Total number of pregnant sows (*n*)	6	6
Weight (kg)	310.7 ± 48.5	316 ± 38.9
Age (years)	3.1 ± 0.4	3.1 ± 0.4
Litter size per sow (*n)*	13.6 ± 0.7	13.2 ± 0.8
Total offspring number (*n*)	80	78
gender (*n_male_/n_female_*)	40/40	36/42
Total number of offspring used (*n*)	12	18
offspring weight (kg)	28.5 ± 4.1	24.2 ± 2.7
Age (days)	74.2 ± 2.7	73.5 ± 2.3
Piglet gender (*n_male_/n_female_*)	6/6	9/9
Body temperature at baseline (°C)	37.6 ± 0.5	37.5 ± 0.4
Body temperature during hypoglycemia (°C)	35.3 ± 0.6	35.8 ± 0.7

Data are given as mean ± SEM.

## Abbreviations

ACTH	Adenocorticotropic hormone
CON	Control group
DEX	Dexamethasone-treated group
EDTA	Ethylenediamine tetraacetic acid
GCs	Synthetic glucocorticoids
HPAA	Hypothalamic-pituitary-adrenocortical axis
